# Visualisation of the T cell differentiation programme by Canonical Correspondence Analysis of transcriptomes

**DOI:** 10.1186/1471-2164-15-1028

**Published:** 2014-11-27

**Authors:** Masahiro Ono, Reiko J Tanaka, Manabu Kano

**Affiliations:** Immunobiology Section, UCL Institute of Child Health, University College London, 30 Guilford Street, London, WC1N 1EH UK; Department of Bioengineering, Imperial College London, London, SW7 2AZ UK; Department of Systems Science, Graduate School of Informatics, Kyoto University, Yoshida-Honmachi, Sakyo-ku, Kyoto, 606-8501 Japan

**Keywords:** Canonical Correspondence Analysis, Multidimensional analysis, Expression microarray, RNA-seq, Immunological genomic data, T cell differentiation, Classification

## Abstract

**Background:**

Currently, in the era of post-genomics, immunology is facing a challenging problem to translate mutant phenotypes into gene functions based on high-throughput data, while taking into account the classifications and functions of immune cells, which requires new methods.

**Results:**

Here we propose a novel application of a multidimensional analysis, Canonical Correspondence Analysis (CCA), to reveal the molecular characteristics of undefined cells in terms of cellular differentiation programmes by analysing two transcriptomic datasets. Using two independent datasets, whether RNA-seq or microarray data, CCA successfully visualised the cross-level relationships between genes, cells, and differentiation programmes, and thereby identified the immunological features of mutant cells (*Gata3*-KO T cells and *Stat3-*KO T cells) in a data-oriented manner*.* With a new concept, *differentiation variable*, CCA provides an automatic classification of cell samples, which had a high sensitivity and a comparable performance to other classification methods. In addition, we elaborate how CCA results can be interpreted, and reveal the features of CCA in comparison with other visualisation techniques.

**Conclusions:**

CCA is a visualisation tool with a classification ability to reveal the cross-level relationships of genes, cells and differentiation programmes. This can be used for characterising the functional defect of cells of interest (e.g. mutant cells) in the context of cellular differentiation. The proposed approach fits with common hypothesis-oriented studies in immunology, and can be used for a wide range of molecular and genomic studies on cellular differentiation mechanisms.

**Electronic supplementary material:**

The online version of this article (doi:10.1186/1471-2164-15-1028) contains supplementary material, which is available to authorized users.

## Background

Analysis of mutant phenotypes has been the major means to reveal gene functions in molecular biology [1]. Currently, in the era of post-genomics, it is anticipated to translate mutant phenotypes into gene functions based on high-throughput data [2]. In immunology, mutant phenotypes have to be translated into gene functions, while taking into account the classification and functions of many interrelated, immune cell subsets, each of which shows dynamic changes in gene expression depending on its differentiation and activation status. This issue is now being recognised in molecular immunology [3], and new methods are required to be developed, in order to understand, based on high-throughput data, the features of cells from mutants in the context of well-characterised differentiation programmes.

In fact, with the expansion of the immune cell classification and the number of available mutant strains, immunological data are becoming more and more *multidimensional* (i.e. many experimental groups), and each measurement can be *high dimensional* (e.g. many genes). In addition, it is common in immunological genomic data that the number of experimental groups is larger than that of replicates (typically duplicate or triplicate as in the Immunological Genome Project [4, 5]), because of large numbers of experimental groups. Thus, it is a major and unique problem in immunology that multidimensionality (of phenotypes) further complicates the famous problem of high dimensionality (of genes) in transcriptomic analysis [6].

In order to analyse such multidimensional data across different experiments, currently the gene signature approach is commonly used in immunology. *Signature* is defined by the characteristic expression of a set of genes in a particular cell subtype [3, 7–10]. However, when multiple subsets are simultaneously analysed, the signature approach is not sufficient by itself and can be misleading, because different signatures can be highly correlated to each other. Thus, the overuse of multiple signatures may further complicate the problem of multidimensionality, and different gene signatures should be properly compared and analysed considering their interrelationships and multidimensionality. Principal Component Analysis (PCA) can provide a useful insight to such a multidimensional problem, but PCA primarily visualises the overall structure of the whole dataset, where uninteresting effects (e.g. between-experimental variations, outliers) can often dominate those of interest [11, 12]. Gene network analysis is often used for the functional analysis of transcriptomic data, and can provide powerful tools for the cross-analysis of multiple datasets [13, 14]. This type of approaches, however, focuses on associations between gene profiles of cells and particular processes within the framework of gene networks, which are usually dependent on annotation database or literature-extracted information [13, 14]. These dependencies are not suitable for investigating totally new and unknown pathways, or examining common, but incorrect hypotheses. Thus, it is hoped to develop a data-oriented method that reveals the cross-level relationships of genes, cells, and multiple differentiation programmes in a transparent manner.

In this study, we have adapted Canonical Correspondence Analysis (CCA) to cross-analyse a transcriptomic dataset of interest (response data) and another transcriptomic dataset (explanatory data) that defines cellular differentiation programmes. CCA measures and visualises similarities (i.e. correlations) between elements across three different levels: genes, cells, and differentiation programmes. Mathematically, CCA uses linear regression and singular value decomposition (SVD), and thereby identifies the linear combinations of explanatory variables that maximise the dispersions of samples in response variables [15]. Thus, CCA effectively deals with the complexity of immunological genomic data in terms of cell subsets and functions analysed. This type of complexity is defined as *multidimensional* in non-biomedical disciplines such as ecology and sociology, and accordingly, *multidimensional analyses* including CCA have developed and widely used in these areas [16, 17]. We recently reported the first adaptation of CCA to microarray data (designated as *CCA on microarray data*, *CCAM*) to visualise the cross-level relationships between pathological and physiological processes for addressing haematological problems [11]. In the current study, we have further extended and developed the use of CCA, so that it effectively analyses a common immunological problem: to identify the functional defect of mutant cells.

We have analysed transcriptomes of CD4^+^ T cells for T cell differentiation in this study. It is known that CD4^+^ T cells, upon antigenic stimulation, differentiate into functionally distinct T cell subsets including interferon-gamma (IFN-γ)-secreting helper T cell-1 cells (Th1), interleukin (IL)-4-secreting Th2, IL-17-secreting Th17 cells, and Foxp3-expressing regulatory T cells (Treg), depending on the cytokine and morphogen milieu [18]. The lineage-specific transcription factors have been identified for each T cell subset: T-bet for Th1, GATA3 for Th2, RORγt for Th17, and Foxp3 for Treg [18–20]. Accordingly, the expression of cytokines and these transcription factors has been commonly used for determining the identities of T cells in terms of their differentiation. On the other hand, recent advances in genomics have revealed that cellular differentiation is not governed by a few dedicated transcription factors, but depends on the activities of multiple transcription factors, almost all of which are expressed and used for other differentiation programmes [21]. Thus, transcriptomic analysis is expected to provide better solutions for fully characterising, and elucidating the identity of, immune cell subsets.

## Results

### Overview of CCA methodology

**Figure 1:**
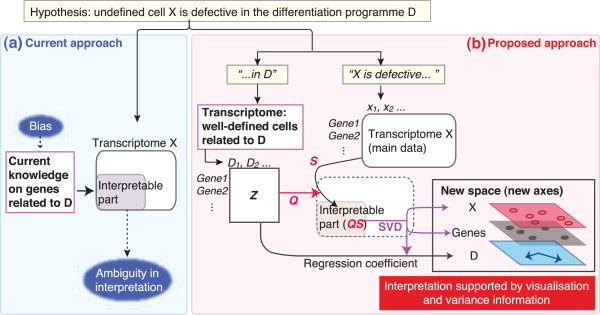
**Delineation of the proposed approach.** Delineation of **(a)** current and **(b)** proposed approaches for studies using transcriptomic analysis. Suppose that the hypothesis for transcriptomic experiment is that cell subset X is defective in the differentiation programme D. **(a)** Typical approach in immunological studies using transcriptomic analysis. Cell subset X and its controls are analysed by microarray analysis or RNA-seq. Note that the interpretation of the results of data analysis is made essentially by “current knowledge,” where considerable arbitrariness and bias can be introduced. **(b)** Proposed approach using Canonical Correspondence Analysis (CCA). The original hypothesis is decomposed into two parts, “*cell subset X is defective…*” and “*…in the differentiation programme D*,” based on which two transcriptomic datasets are prepared. Note that the same genes must be used in both matrices ***Z*** and ***X***. *X* is standardised (S), and projected onto ***Z*** using a projection matrix ***Q***. Thus, the projected space ***QS*** is the interpretable part of the main data ***X***. SVD is applied to ***QS***, producing sample and gene scores (‘X’ and ‘Gene’ in the new space). Differentiation programmes are visualised as regression coefficients between ***Z*** and the new axes. The results are visualised as a triplot that show relationships between cell subset X, genes, and differentiation programmes. The visualisation process ensures the transparency of the interpretation.

In this study, CCA has been adapted to analyse transcriptomic data and thereby specifically identify which differentiation programme (D) is disturbed in undefined cell subset X (e.g. T cells from some KO mice, Figure 1). Currently, the typical approach for this problem is to analyse the transcriptomes (***X***), and interpret the results of the analysis by current knowledge (e.g. the literature and annotation databases) on the genes that are related to D (Figure 1a). On the other hand, the proposed approach first decomposes the original hypothesis into two parts, “*cell subset X is defective…*” and “*…in the differentiation programme D*,” based on which two transcriptomic datasets are prepared. Next, CCA is applied to the transcriptomic data ***X***, using the dataset for D (***Z***, or resource dataset) as explanatory variables. The standardised matrix of ***X***, ***S***, is projected onto ***Z***, and thus, the projected space ***QS*** is the interpretable part of the main data ***X*** by the explanatory variables. SVD is applied to ***QS***, producing sample and gene scores (X and Gene in the new space). Differentiation programmes are visualised as regression coefficients between ***Z*** and the new axes. These results are visualised as a triplot that show relationships between cell subsets, genes, and differentiation programmes, facilitating hypothesis-generation based on the interpretation of data in a data-oriented manner (Figure 1b).

CCA was originally developed by ter Braak for analysing data of fish species in various locations in the ocean in the context of ‘environmental gradients’ (e.g. ion concentrations), in order to visualise the relationships between the geographical location (site), fish species, and environmental gradients in the ocean [15, 22]. In our method, we define gene expression as the amount of transcripts occurs at each gene (corresponding to ‘site’ by ter Braak), and assume that transcripts are measured at those sites by microarray or RNA-seq experiments for cellular phenotypes (corresponding to ‘species’). Transcriptomes of well-defined, differentiated cells represent differentiation programmes (corresponding to environmental gradients), and the gene expression profiles of those cells are used as explanatory variables. Mathematically, CCA projects the main dataset onto explanatory variables, and perform SVD in the projected space using the algorithm of Correspondence Analysis (Figure 1b), which is a weighted PCA in the chi-square metric [22].

While visualisation is the primary strength of CCA, we have developed a new approach for characterising and classifying samples using CCA by introducing *differentiation variable* as explanatory variable, which is equivalent to environmental gradient by ter Braak [15, 22]. Here we assume that a cell phenotype X can change into the one of another cell phenotype Y. Considering that explanatory variables are used for regression, differentiation variable ***d*** is defined as the responses of a set of transcripts when a cell changes its phenotype from X to Y, ***d*** = ***μ***_***y***_ – ***μ***_***x***_ , using mean gene expression profiles of X and Y, ***μ***_***x***_ and ***μ***_***y***_, which is equivalent to environmental gradient by ter Braak [15, 22]. Thus, within-group variations in the explanatory data are not considered in CCA, and the data needs to have sufficiently large between-group variance and small within-group variance, as typically seen in immunological genomic data. When only one differentiation variable is used as explanatory variable, CCA provides one-dimensional solution, which can be used as a new scoring system for the association of genes and samples with the differentiation programme.

We have analysed two immunological problems using two sets of transcriptomic data in this study. In each analysis, we first examine the visualisation ability of CCA and elaborate how CCA results can be interpreted. Next, we compared the classification ability of CCA with other classification methods. Table 1 summarises the characteristic of datasets used in this study.

**Table 1:** **Datasets used in this study**

GEO accession	Summary	Number of classes	Number of replicates	Platform	GEO accession (platform)	Citation
GSE14308	Microarray data of Th subsets (in vitro-generated Th1, Th2, Th17, and iTreg) and freshly sorted Treg and naïve T cells	6	2	Affymetrix GeneChip	GPL1261	[23]
				Mouse Genome		
				430 2.0 Array		
GSE20898	RNA-seq data of (in vitro-generated Th1, Th2, Th17, and iTreg) from Gata3-KO and WT	8	2	Illumina Genome	GPL9250	[24]
				Analyzer II		
GSE21670	*Stat3*-KO and WT T cells under various culture conditions	8	2	Affymetrix GeneChip	GPL1261	[25]
				Mouse Genome		
				430 2.0 Array		

### Exemplary analysis (1): Identify the major effect of Gata3-deletion on T cell differentiation

**Figure 2:**
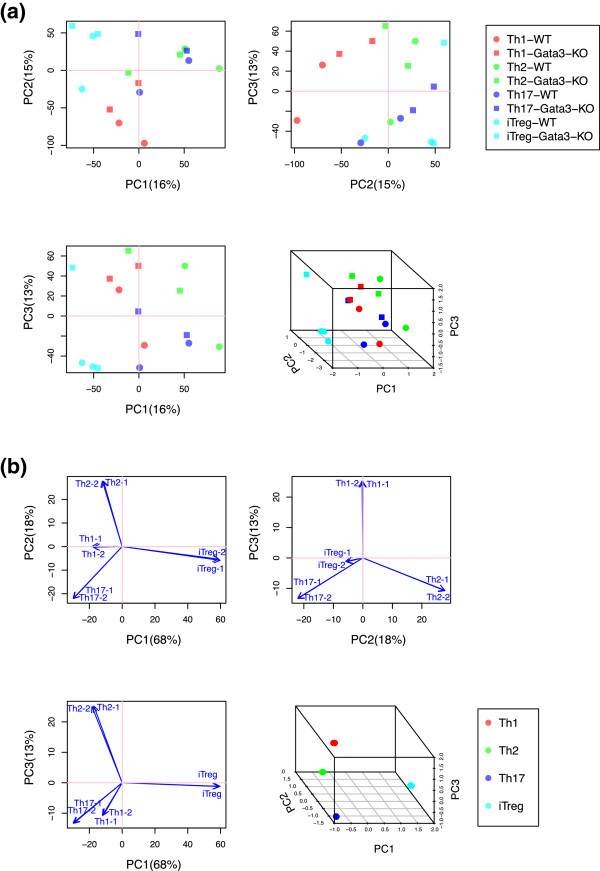
**PCA sample scores of the datasets that were used in CCA analysis.** PCA was applied to **(a)** the Gata3 dataset and **(b)** the Th dataset. Sample relationships (sample scores) of the first 3 axes are shown. Sample scores in 2D plots **(b)** are deliberately shown by arrows, in order to emphasise that these samples correspond to the explanatory variables that are shown by blue arrows in Figure 3. Percentage indicates that of the variance accounted for by the eigenvalue of the axis.

In this analysis, we analysed an RNA-seq dataset of *Gata3*-KO and WT T cells including Th1, Th2, Th17, and iTreg (GSE20898 [24], designated as the *Gata3 dataset*) and a microarray dataset that analysed the same Th subsets from WT mice (GSE14308 [23], designated as the *Th dataset*). The purpose of this analysis is to identify which Th differentiation programme is most disturbed by the deletion of the *Gata3* gene. The results of PCA using these datasets are shown in Figure 2, confirming good separations of sample classes, although they do not provide insights into the function of Gata3.

#### Sample and gene score analysis using CCA triplot

**Figure 3:**
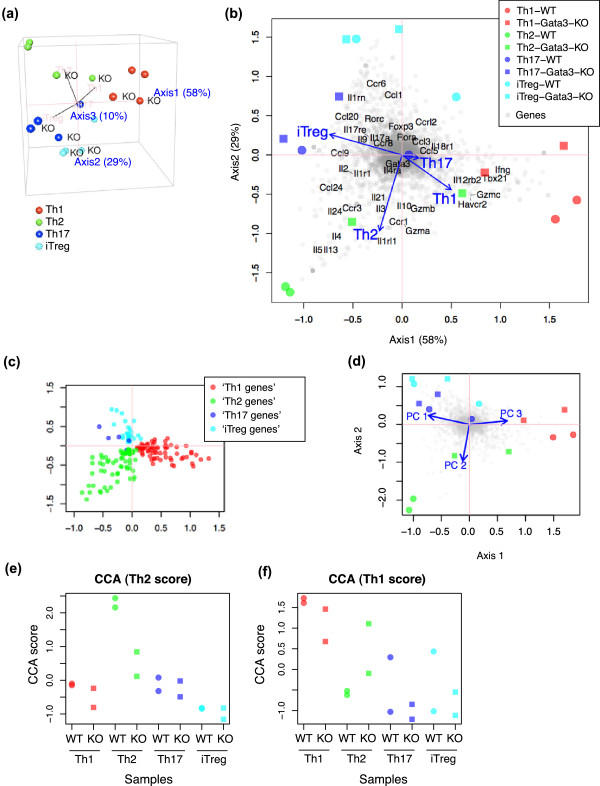
**CCA results using the Gata3 dataset for the Th differentiation programmes.** CCA was applied to the Gata3 dataset, using the microarray dataset that analysed Th1, Th2, Th17, and iTreg (the Th dataset) as explanatory variables for the Th differentiation programmes. **(a)** Sample relationships in the first three axes. The Th differentiation programmes are shown by black lines (pink texts). **(b)** CCA triplot of *Gata3*-KO and WT samples (red, green, blue and cyan closed circles and squares), genes (grey closed circles), and the Th differentiation programmes (blue arrows). **(c)** Gene plot of the CCA solution in **(a)** and **(b)**, showing Th-specific genes only. **(d)** CCA triplot using PCA gene scores (PC1-3) of the Th dataset as explanatory variables. **(e, f)** CCA sample scores using **(e)** Th2 and **(f)** Th1 differentiation variables.

CCA was applied to the Gata3 dataset, using the Th dataset as explanatory variables. CCA clustered Th1, Th2, Th17, and iTreg RNA-seq samples in the first 3 axes (Figure 3a). The main features of the sample relationships are, however, mostly contained in the first 2 axes, which occupied 87% of the constrained inertia. CCA triplot shows the correlations between genes, cell samples, and differentiation programmes (Figure 3b). In other words, the more correlated, the nearer the components are positioned on the map [11, 22]. Biplot values of the CCA result in Figure 3b (shown by arrows) were different from the sample scores of PCA of the Th dataset in Figure 2b, indicating that CCA has provided a unique solution. The Th1 and Th2 differentiation programmes (explanatory variables) were correlated with their corresponding RNA-seq samples (Figure 3b). All T cell subsets from the RNA-seq data and their specific genes were associated by CCA (Figure 3b and c; note that these two plots are comparable; see Methods for Th-specific genes).

Remarkably, *Gata3*-WT Th2 cells had high negative values in Axis 1 and 2, with which well-known Th2 genes including *Il4*, *Il5*, and *Il13* were associated, while *Gata3*-KO Th2 cells did not (Figure 3b). Although Th1 cells showed a difference in *Gata3*-KO and WT, other Th cells did not show any obvious difference, suggesting that the effect of the Gata3-deletion was more obvious in Th2 and Th1 differentiation.

#### CCA using PCA scores as explanatory variables

Next, we applied CCA to the Gata3 dataset, using PCA gene scores of the Th dataset as explanatory variables, in order to obtain further insights on the CCA results. The sample and gene relationships were mostly similar between the CCA results using the original explanatory variables and PCA gene scores (Figure 3b and d), presumably because PC1, 2, and 3 contained more than 98% of the total variance. Biplot values of differentiation programmes (arrows) in Figure 3d can be mostly explained by the linear combinations of Th sample vectors in the PCA result in Figure 2b: PC1, 2, and 3 represent the difference between iTreg and all others, between Th2 and Th17, and between Th1 and both Th2 and Th17, respectively. This result confirms the linearity of CCA, which dimensions are intentionally defined as linear combinations of the explanatory variables [17].

#### CCA results by differentiation variables

In order to further examine the correlations between samples and the Th1 and Th2 differentiation programmes, we analysed the Gata3 dataset using corresponding differentiation variables. Using a Th2 differentiation variable, the CCA solution showed that WT Th2 cells had the highest scores, while KO Th2 and other Th cell populations had low scores (this CCA sample score is designated as the *Th2 score*; Figure 3e). On the other hand, CCA analysis using the Th1 differentiation variable showed that, although WT Th1 cells had the highest scores, there was only a small difference between WT and KO Th1 cells (designated as the *Th1 score*; Figure 3f). Percent explained variance (precisely, *inertia*; see Methods) was similar between two analyses (1.1% and 1.4% for Th2 and Th1 scores, respectively). Thus, even considering that the overall dispersion of the Th1 score was approximately 30% larger, the difference between WT and KO in the Th2 score was most remarkable in these two analyses.

### Comparison of CCA with other classification methods using the Gata3 dataset

**Figure 4:**
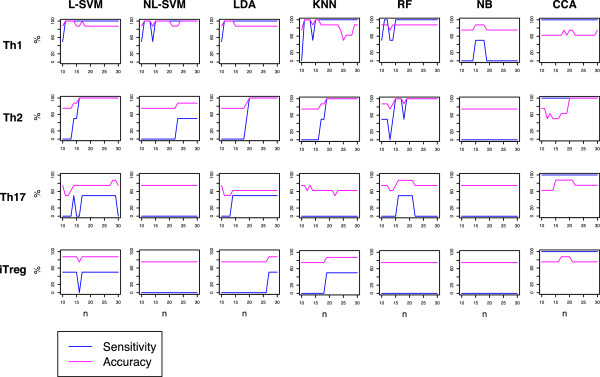
**Comparison of CCA with other classification methods using the Gata3 dataset.** The classification ability of CCA was compared with other classification methods. The Th dataset was used as a training data (explanatory variables for CCA), and WT data from the Gata3 dataset was used as a test data. Sensitivity and accuracy of those methods are plotted for each T cell subset (shown on the left side), using various numbers of genes (*n*; between 10 and 30). The numbers of condition positive (‘correct’ Th samples) and condition negative (all other samples) are two and six, respectively, in all the analyses.

Using the Gata3 and Th datasets, the classification ability of CCA using a differentiation variable was compared with other classification methods: linear and non-linear support vector machines (L- and NL-SVMs), linear discriminant analysis (LDA), K-nearest neighbor (KNN), Naïve Bayes (NB), and Random Forest (RF). The Th dataset was used as a training data (a resource dataset for CCA), and WT data from the Gata3 dataset was used as a test data. Thus, we addressed how efficiently those classification methods identify Th transcriptomes from RNA-seq data, based on those from microarray data. Table 2 shows the results of these analyses. Using various numbers of genes, CCA had high sensitivities (100%) and good accuracies (Figure 4). Thus, CCA outperformed, or at least was equivalent to, other classification methods.

**Table 2:** **Classification ability of CCA and other classification methods by Gata3 and Th datasets**

Sensitivity (%)							
Th cells to identify	L-SVM	NL-SVM	LDA	KNN	RF	NB	CCA
Th1	100	0	100	100	100	0	100
Th2	100	0	100	100	100	0	100
Th17	0	0	50	50	0	0	100
iTreg	50	0	50	50	0	0	100
**Accuracy (%)**							
**Th cells to identify**	**L-SVM**	**NL-SVM**	**LDA**	**KNN**	**RF**	**NB**	**CCA**
Th1	87.5	75	87.5	100	100	75	87.5
Th2	100	75	100	100	100	75	100
Th17	75	75	62.5	87.5	62.5	75	75
iTreg	75	75	87.5	87.5	75	75	75

Training and test data were the Th dataset and the Gata3 dataset, respectively. The number of feature used was 100.

### Exemplary analysis (2): Identify the functional defect of Stat3-KO T cells in T cell differentiation

In this section, we analysed a dataset of *Stat3*-KO and WT T cells in various culture conditions (GSE21670 [25], designated as the *Stat3 dataset*) and the Th dataset (Table 1). Previous reports showed that WT T cells differentiated into Th17 in the presence of IL-6, while *Stat3*-KO T cells did not [25]. Thus, the purpose of the analysis is to address whether CCA and other methods can reveal that Th17 differentiation was most disturbed in *Stat3*-KO T cells. In addition, we examined whether CCA can reveal hidden associations between genes, samples, and differentiation programmes.

#### Analysis of the Stat3-KO dataset by conventional approaches

First, we used the *signature* approach with hierarchical clustering and PCA as competing methods, in order to address these problems. Gene signatures for Th1, Th2, Th17, and iTreg were generated using the Th dataset by an empirical Bayes test. Hierarchical clustering showed that only the iTreg signature clustered WT T cells cultured with IL-6, whether with or without TGF-β (hereafter designated as WT.IL6.TGFβ and WT.IL6, respectively; Additional file 1), which are known to differentiate into Th17 cells [25]. This result, however, is difficult to be immunologically interpreted. Next, PCA was applied to the Stat3 dataset (Additional file 2). The first 3 axes occupied 56% of total variance, but sample relationships in these 3 axes were apparently not immunologically meaningful. Thus, both the signature approach and PCA failed to reveal the features of *Stat3* KO T cells.

#### Identify the functional defect of Stat3-KO T cells in T cell differentiation by CCA

**Figure 5:**
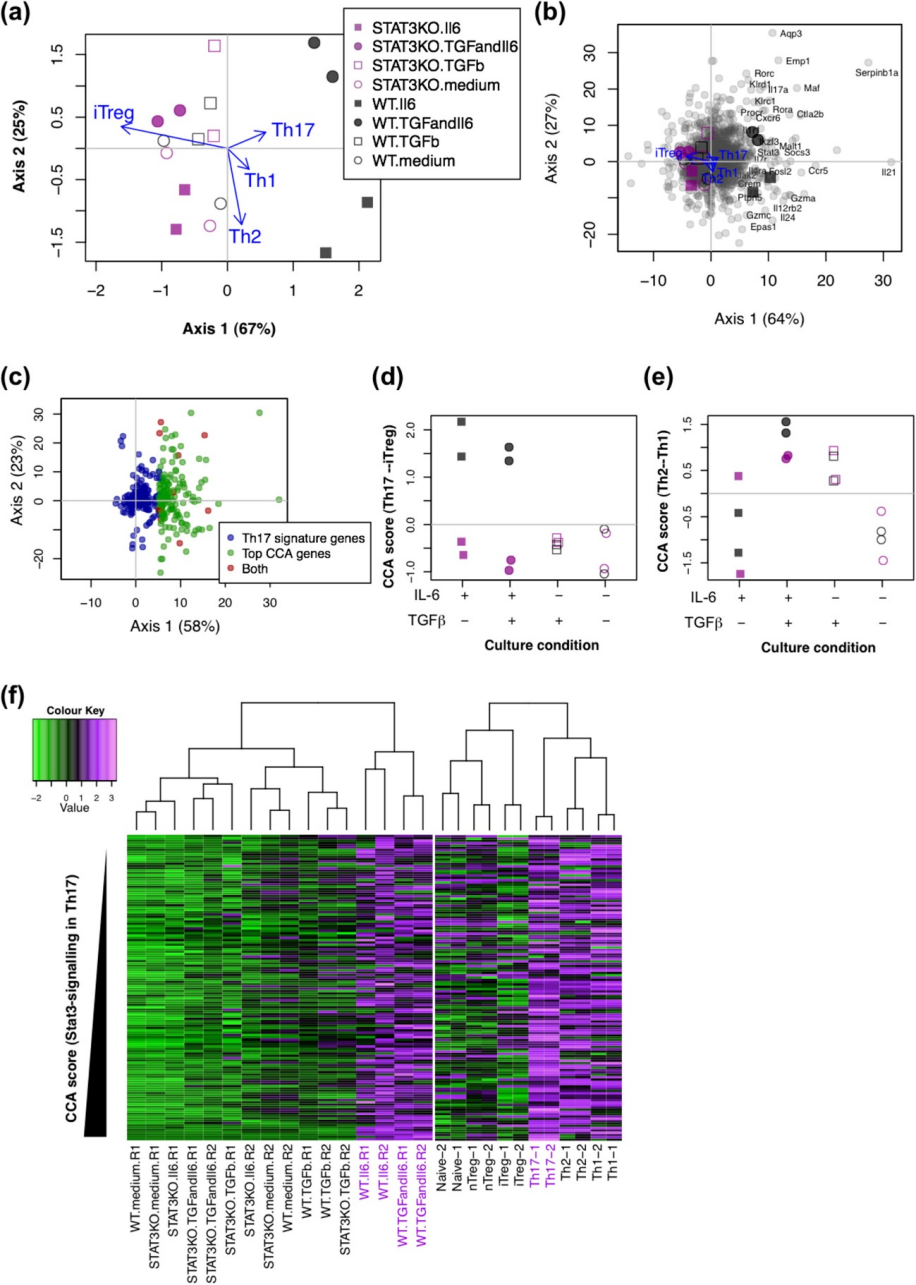
**Identify the T cell differentiation programmes that are disturbed in** ***Stat3*** **-KO by CCA.** The Stat3 dataset was analysed by CCA using the Th dataset as explanatory variables. **(a)** CCA biplot showing the relationships between samples (see legend) and Th differentiation programmes (arrows). Percentage indicates that of the variance accounted for by the inertia of the axis. **(b)** CCA triplot showing samples (see legend in **(a)**), Th differentiation programmes (arrows), and genes (small grey closed circles). **(c)** Gene plot of the CCA solution in **(a)** and **(b)** showing the ‘Th17-signature genes’ and the CCA top-ranked genes (2% top genes in Axis 1) only. Genes in the intersection of these two gene lists are shown as ‘Both’ in the legend. **(d, e)** CCA sample scores using **(d)** Th17/iTreg and **(e)** Th2/Th1 differentiation variables. Differentially expressed genes by the explanatory dataset (the Th dataset) were selected by false discovery rate (FDR) <0.01, and fold change (top/bottom 1%) in the comparison of Th2 and Th17, or that of Th1 and iTreg. **(f)** Heatmap analysis of the top-ranked genes in **(c)**. Gene expression of those genes in the Stat3 dataset (left) and that in the Th dataset (right) were separately analysed by heatmap analysis, while clustering column (samples) only. Genes were ordered according to the CCA Axis 1 score. See Colour Key for expression levels.

CCA was applied to the Stat3 dataset, using the Th dataset as explanatory variables (Figure 5). In this part, we mainly examine the visualisation ability of CCA, while elaborating how CCA results can be interpreted.

#### CCA sample score analysis

Axis 1 occupied 64% of the constrained space, and explained major variations in this analysis. WT.IL6 and WT.IL6.TGFβ showed high scores in Axis 1, while all other samples including *Stat3* KO T cells with the same conditions were negative. Among the Th differentiation programmes (arrows in Figure 5a), Th17 differentiation programme showed the highest positive correlation with Axis 1, and the iTreg differentiation programme strongly and negatively correlated with Axis 1 (Figure 5a).

Thus, CCA succeeded in identifying the known fact that WT.IL6 and WT.IL6.TGFβ differentiated into Th17 cells, while *Stat3* KO T cells failed [25]. In addition, the Th1 differentiation programme showed the second most positive correlation with Axis 1, and thus, with WT.IL6 and WT.IL6.TGFβ (Figure 5a, see Discussion).

#### CCA gene score analysis

Based on the analysis above, genes with high scores in Axis 1 (hereafter designated as top-ranked genes) were presumably related to either or both of Th17 differentiation and Stat3 signalling. In fact, they were enriched with Th17-related genes: (1) well-known Th17 genes such as *Il21*, *Il17a, Klrd1, Stat3*, *Fosl2*, *Serpinb1a*, *Rora*, *Rorc,* and *Maf* were high positive ([26]; Figure 5b, Additional file 3); and (2) more than 70% of Th17 signature genes by Yosef *et al.*[26] had positive values in Axis 1 (Figure 5c). In addition, many of these CCA top-ranked genes were related to either or both of Th17 differentiation and Stat3 signalling by preceding studies (Additional file 3).

#### CCA results by differentiation variables

In order to further address which differentiation programme is most correlated with the defect of *Stat3*-KO T cells, we applied CCA to the Stat3 dataset using Th17/iTreg and Th2/Th1 differentiation variables (i.e. the difference in gene expression between Th17 and iTreg, and between Th2 and Th1, respectively; Figure 5d, 5e). The CCA solution using a Th17/iTreg differentiation variable showed that WT.IL6 and WT.IL6.TGFβ had higher scores (i.e. more Th17-ness) (Figure 5d). Th2/Th1 differentiation variable did not provide meaningful results (Figure 5e). Top-ranked genes in Axis 1 (2%, Figure 5c) were highly expressed in WT.IL6 and WT.IL6.TGFβ (Figure 5f, left panel) and in Th17 cells (Figure 5f, right panel) by a heatmap analysis. Collectively, CCA revealed the relationship between Stat3-KO and WT T cells and those different programmes in a data-oriented manner.

### Comparison of CCA with other classification methods using the Stat3 dataset

**Figure 6:**
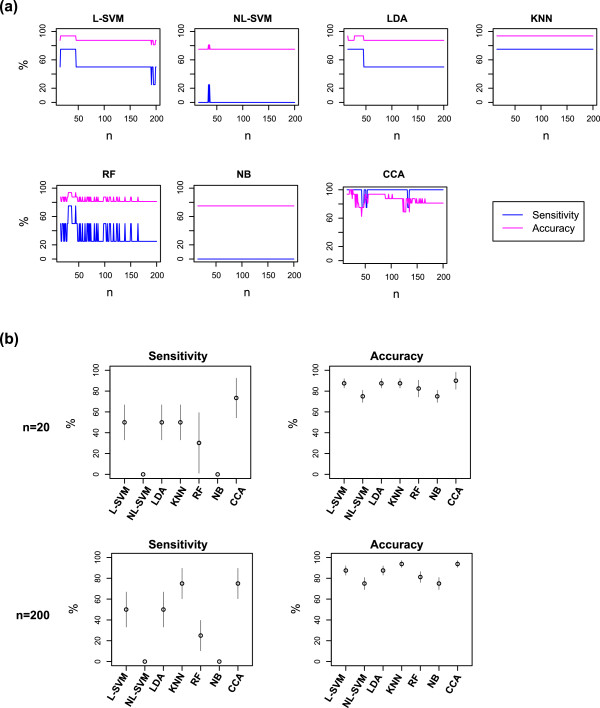
**Comparison of CCA with other classification methods using the Gata3 dataset.** The classification ability of CCA was compared with other classification methods. The Th dataset was used as a training data (explanatory variables for CCA), and WT data from the Stat3 dataset was used as a test data. **(a)** Sensitivity and accuracy of those methods are plotted, using various numbers of genes (*n*; between 10 and 200). The numbers of condition positive (Th17 differentiated cells) and condition negative (all other samples) are four and twelve, respectively, in all the analyses. **(b)** Test dataset was resampled using a jackknife approach, and the classification methods were compared for sensitivity and accuracy. The number of genes used was either 20 (upper panels) or 200 (lower panels). Error bar indicates 95% confidence interval.

In this last section, the classification ability of CCA was compared with other classification methods using Th dataset as a training data (a resource dataset for CCA), and the Stat3 dataset was used as a test data. We addressed how efficiently those classification methods identify Th17 differentiated T cells in the Stat3 dataset, based on the resource dataset. Using various numbers of genes, CCA had high sensitivities and accuracies and outperformed, or at least was equivalent to, other classification methods (Figure 6a). This result was confirmed using a jackknife method, where multiple test datasets were generated by leave-one-out from the Stat3 dataset (Figure 6b).

## Discussion

### Methodological considerations

In typical immunological hypotheses, genes and cell phenotypes are correlated with immunological processes, and accordingly, genomic data are often ‘filtered’ for the immunological processes of interest (corresponding to the interpretable part of X in Figure 1). This is typically achieved by selecting a set of genes based on the analysis of experimental data, annotation database, or literature-extracted information on protein-protein regulation network [13, 14, 27]. Here CCA uses a linear regression to identify the interpretable part of the main data (*constrained space*) and finds a solution within the constrained space, while ignoring the non-interpretable part of the data (*unconstrained space*) [15, 22]. Thus, CCA is more transparent in its operation, and less dependent on the literature, comparing with the methods above, and thus can be used for the experimental study that analyses rare phenomenon or addresses controversial hypotheses. In addition, with the regression approach, CCA is implemented with the basic structure of hypothesis-oriented study: hypothesis can be usually decomposed into two parts, the main part and its biological context, which are analysed by CCA as the main and explanatory data (Figure 1b). This may explain why CCA worked more efficiently than PCA, which cannot incorporate the layered structure of biological hypothesis. In addition, CCA worked more efficiently than PCA, presumably because CCA analyses the constrained space, so that the result that CCA produces is biologically meaningful. On the other hand, there are drawbacks of the proposed method. Explanatory variables cannot include highly correlated variables, because they are used for regression. In addition, sample variations in the resource dataset that defines explanatory variables are ignored. Therefore, CCA is primarily suitable for the analysis of undefined cells in relation to the differentiation programmes that are represented by well-defined cell subsets. It seems that immunologists empirically know that their data have small within-group variance and large between-group variance (c.f. Figure 2b), and most of immunological genomic datasets have no more than duplicate or triplicate (e.g. Immunological Genome Project [4, 5]). Using such data, PCA gene scores may serve well as explanatory variables for CCA as shown in Figure 3d, if the dimensionality of the data can be reduced in a biologically meaningful way.

CCA had high sensitivities for identifying correlated samples in the cross-analysis of two datasets (Figure 4 and 6). This may be because CCA identifies and analyses only the part of the main data that can be explained by another dataset (i.e. constrained data [22]). In addition, Correspondence Analysis, an underlying algorithm of CCA, primarily concerns correlations: it assigns high negative/positive values to genes that show high correlations to specific samples, while allocating low negative/positive values to non-correlated elements [16, 17, 22]. Thus, presumably CCA is efficient in identifying the cell samples that show high correlations to a differentiation variable. On the other hand, CCA showed relatively lower accuracies comparing with its remarkably high sensitivities (Figure 6). In fact, CCA is not designed to deliberately discriminate groups, as other classification methods are [28], but aims to measure the distance (correlations) between samples in a space with reduced dimensionality, while maximising their overall dispersions [17].

The proposed method assumes that gene expression is the measurement of transcripts at each gene (site), which represents a local ‘activity’ for a cell phenotype, and the total set of those transcripts (i.e. transcriptome) collectively shapes the cellular phenotype. Thus, CCA uses two matrices with genes in rows and samples in columns, and the genes must be the same between the two matrices (Figure 1b). Although it is often recommended to have samples (as observations) in rows and genes (as features) in columns so that sample size is considered [29], it is in fact a common practice to apply PCA to a matrix with genes in rows and samples in columns, in order to analyse genes in the sample space [30]. This is geometrically a sensible way to apply PCA to transcriptomic data, considering that, by definition, the number of principal components cannot exceed the number of samples or genes, either of the smaller ones [31], and that PCA is a procedure to reposition the origin at the centroid of the points in a multidimensional space and then to rotate the coordinate axes in such a way as to satisfy the maximal variance property [32]. In other words, when the number of genes is much larger than that of samples, the coordinate axes can be meaningfully rotated only in the sample space. Thus, our method analyses the sample space that is constrained by differentiation programmes as explanatory variables, providing sample and gene spaces with new coordinate axes. The comparison of those two spaces has been extensively studied as *the theory of the duality diagram*, which is implemented in the CRAN package *ade4*[33].

### Immunological considerations

In the original report of the dataset GSE20898, the data were mostly mined by Venn diagram analysis, and the authors emphasised Gata3-mediated gene regulations in all the analysed T cell subsets (Th1, Th2, Th17, and iTreg) [24]. On the other hand, CCA identified that the deletion of *Gata3* had the largest impact on Th2 differentiation, and also suggested that it had some effects on Th1 differentiation. In fact, Gata3 has been closely linked to Th2: Th2 differentiation is totally abolished *in vitro* and *in vivo* by the conditional deletion of Gata3 [18]. Interestingly, *Gata3*-KO Th2 cells were closer to Th1 cells than WT Th2 cells in our analysis (Figure 3b), which may be, at least partly, related to the increase of Th1-specific genes including *Tbx21* and *Il12rb2* in *Gata3*-KO Th2 cells [24]. The dysregulation of Th1 genes in *Gata3*-KO Th2 cells may be due to the opposing interaction between Tbet and Gata3 [34]: *Gata3*-KO T cells may have an aberrant activity of Tbet, which also explains the possible effect of Gata3-deletion on Th1 differentiation (Figure 3f). Thus, CCA has provided a useful bird’s eye view on the Gata3 dataset. Further studies on the time course of differentiating Th1 and Th2 transcriptomes, using WT and *Gata3*-KO, may reveal how this differentiation programme is activated and how Gata3-deletion affects the programme.

CCA identified the Th17 differentiation programme as the most disturbed process in *Stat3*-KO T cells. This result is compatible with the findings by Durant *et al.,* which showed that *Stat3* was required for Th17 differentiation by *in vivo* and *in vitro* experiments using *Stat3*-KO T cells [25]. The CCA result also indicated that the most correlated process of *Stat3*-KO T cells was the iTreg differentiation programme (Figure 5a). Considering that Th17 and iTreg differentiation are mutually controlled by IL-6 and IL-2, respectively, at the cytokine level, and by RORγ-t and Foxp3, at the transcription factor level [35, 36], *Stat3*-KO T cells may have a stronger tendency to differentiate into iTreg. Interestingly, Durant *et al.* observed that *Stat3*-KO mice produced larger numbers of Treg in experimental colitis than WT mice [25]. In addition, CCA identified that Th1 differentiation was the second most disturbed process in *Stat3*-KO T cells (Figure 5a). In fact, Th1 and Th17 are highly related processes: before the emergence of Th17, Th1 had been thought to cause autoimmune diseases such as experimental autoimmune encephalitis and arthritis, which are nowadays more associated with Th17 [36, 37]. In addition, Stat3 is functionally related to Th1, whether positive or negative: the Th1 cytokine IL-12 activates not only Stat4 but also Stat3 [38]; *Stat3*-KO mice show either enhanced or decreased Th1 response depending on the experimental settings [39, 40]. Recently, the interrelation between Th1 and Th17 has also been studied for their plasticity and stability, confirming their close associations [41]. CCA also showed that WT T cells with IL-6 in the absence of TGF-β were correlated with Th1 genes including *CCR5, Il12rb2* and *Gzma*. IL-6 in the absence of TGF-β is known to less stably induce IL-17A production, and T cells have a more Th1-like phenotype [41].

Immunological studies may become more robust against *confirmation bias* if proper multidimensional techniques are introduced [42]. Confirmation bias is widely known in sociology, politics and psychology, and is defined as the seeking or interpreting of evidence in ways that are partial to existing beliefs, expectations, or a hypothesis in hand [43]. It is the bias behind our research practice, not a statistical bias, being introduced not only by our own nature but also by the peer review process [42, 44]. When analysing complex multidimensional data, researchers can easily pick up small differences between samples in favour of their hypothesis (i.e. confirmation bias), while ignoring the major trends in the data. CCA can fight this bias by visualising the relationships of samples and/or genes and thereby facilitating interpretations with minimal assumptions, as demonstrated in this study.

## Conclusions

The proposed method can be used for revealing the cross-level relationships between genes, samples, and biological processes of interest based on two transcriptomic data. The visualisation of the result that CCA produces is essential, relating undefined cells to known biological processes and genes, and thereby unravelling complex relationships between multiple phenotypes and genotypes. Thus, CCA can provide a platform (triplot) that facilitates the generation and refinement of hypothesis. In addition, CCA can have a high sensitivity for identifying the differentiated cells in a dataset that are similar to the ones in another dataset. These unique features make CCA competitive with other existing methods. The proposed method can be applied to a wide range of biological problems, providing effective solutions for multidimensional problems with multiple phenotypes and functions.

## Methods

### Datasets and data analysis

The datasets used in this study are summarised in Table 1. Computational analysis was performed using Mac OS 10.6.8 and R version 3.0.2. Microarray data were normalised by *rma* of the Bioconductor package, *affy*. RNA-seq data were normalised for RPKM and log2 transformed. The function *dudi.pca* of the CRAN package *ade4* was used for PCA. The function *cca* of the CRAN package, *vegan*, was used for the calculation of CCA.

### Canonical Correspondence Analysis (CCA)

The main transcriptomic data ***X*** ∈ ℜ^*k × p*^ and is composed of the measurements of *p* cellular phenotypes at *k* genes (sites; see text). The j-th phenotype of ***X***, x_j_ = (*x*_*1j*_*x*_*2j*_ … *x*_*kj*_)^T^ is the experiment vector of *k* genes (i.e. transcripts occurred in ‘*k* gene sites’), where T indicates transposed vector. Similarly, ***Z*** ∈ ℜ^*k × q*^ is a matrix for explanatory variables (differentiation variables) and have *k* gene sites and *q* differentiation programmes (do not include replicates). ***Z*** is standardized to mean 0 and variance 1. First, ***X*** is standardised in the chi-square metric by row sums (***r***, i.e. gene expression levels) and column sums (***c,*** samples). Thus, the standardised matrix is ***S*** = ***D***_***r***_^-1/2^ (*1/n****X***– ***rc***^T^) ***D***_***c***_^-1/2^, where *n* is the grand total of expression data, ***D***_***r***_ and ***D***_***c***_ are the diagonal matrices of ***r*** and ***c***, respectively. CCA linearly regresses ***S*** onto differentiation variables ***Z***, by the projection matrix ***Q*** = ***D***_***r***_^1/2^***Z*** (***Z***^T^***D***_***r***_***Z***)^-1^***Z***^T^***D***_***r***_^1/2^, and the constrained (projected) space S* = ***Q S***[17]. Thus, this projection incorporates the weighting of the rows (average gene expression levels) in the diagonal matrix of row masses. Next, CCA finds new axes by assigning numerical values to samples and genes so that the dispersion of samples is maximised [15]. Mathematically, this step is equivalent to singular value decomposition (SVD) of the standardised matrix ***S***[17]. By calculating the SVD of S*, S* = U D_***α***_***V***^T^, where ***U***^T^***U*** = ***V***^T^***V*** = ***I***, and ***D***_***α***_ is the diagonal matrix of singular values in descending order (α_1_ ≥ α_2_ ≥ …). Principal or standard co-ordinates for gene expression scores (actually, the linear combination [LC] scores) and sample scores are ***D***_***r***_^-1/2^***U D***_***α***_*or****D***_***r***_^-1/2^***U,*** and ***D***_***c***_^-1/2^ ***V D***_***α***_ or ***D***_***c***_^-1/2^ ***V****,* respectively [17] (refer to [33] for the relationship between these two co-ordinates). The LC scores ***D***_***r***_^-1/2^***U***, however, are in fact linear combinations of differentiation variables [45, 46]. Thus, in order to relate gene scores to the samples, gene scores are defined by weighted average scores (WA scores), which are obtained by projecting ***S*** onto the sample scores, namely ***S V D***_***α***_ , or ***S V***[45, 46]. For the visualisation of differentiation variables in CCA result, the biplot values of differentiation variables (arrows in triplots) are calculated as weighted correlation coefficients (regression coefficients) of original differentiation variables ***Z*** and the new co-ordinate axes, or more precisely, the LC scores, [45, 46] (Figure 1).

When only one differentiation variable is used, SVD does not have to be used, and CCA regresses the main data onto the differentiation variable, and assigns numerical values to samples and genes so that the dispersion of samples is maximised [15], providing a one-dimension solution. This is exactly the same definition of the Japanese version of Correspondence Analysis, *Hayashi’s quantification method III*, which has been extensively used for creating scoring systems for qualitative data mainly in psychology and sociology [16]. Similarly, the sample and gene scores of CCA result can be used as a new scoring system for genes and samples, as shown in this study.

*Inertia* is the sum of total Pearson χ^2^ divided by the total sum, and is the measurement of variations in CCA and plays the same role as the total variance in PCA. CCA decomposes total inertia *I*_*T*_ into two parts, constrained inertia, *I*_*C*_, and unconstrained inertia, *I*_*U*_ = *I*_*T*_ - *I*_*C*_.%*Explained* is defined as *I*_*C*_*/ I*_*T*_ and represents how much of the information in the original data is retained in CCA solution [11].

### Gene lists and gene signature

Th-specific genes and Gata3-regulated genes referred to the lists provided by Wei *et al.*[24]: a lineage-specific gene was defined to have a RPKM (reads per kilo base of exon model per million mapped reads) ≥5 and should be 2-fold greater than in other lineages; Gata3-regulated genes were defined as differentially expressed genes between *Gata3*-KO and WT Th2 cells. Th1-, Th2-, Th17-, and iTreg-specific genes were 91, 90, 7, and 43 genes, respectively, and Gata3-regulated genes were 623 genes [24]. *Th17 signature genes* (Figure 5c) referred to the ranked gene lists for Th17 regulators by Yosef *et al.*[26]. *Gene signatures* of Th subset were selected using GSE14308 (Th dataset) by Gene Set Enrichment Analysis (GSEA) [13, 14, 27]: genes were filtered by an Empirical Bayes test (*limma*[47]) with FDR <0.01 and fold change >1, and GSEA was performed using these genes and the C7 collection of MSigDB (immunologic signatures) for each Th subset (in comparison to all other Th samples) with 1000 permutations of gene sets. The top 50 genes by GSEA were used for hierarchical clustering by the function *heatmap.2* of the CRAN package, *gplots*, using the complete-linkage clustering using the Euclidean distance.

### Machine learning methods and classifications

In order to apply machine learning methods, training and test data were cross-normalised using an empirical Bayes approach of the Bioconductor package *virtualArray*[48] or a rank normalisation method of the package *demi*[49]. Each Th subset was compared to all other Th subsets using the Th dataset as a training data, and those models were tested for the Stat dataset. Linear and non-linear (radial) support vector machine (SVMs) and Naïve Bayes classifier were performed using the CRAN packages, *e1071*[50]. For classifying the Th17 differentiation by the non-linear SVM, various gamma parameter and cost values were extensively tested, using the tuning function of *e1071*. In the analysis in Figure 6, gamma parameter was set to 1 divided by the number of genes, and the cost was set to 1, using the C-classification method. K-nearest neighbour (KNN) was applied using the CRAN package *class*[51]*,* and the number of neighbours was set to be 2. Linear and diagonal discriminant analyses were performed using the CRAN package, *sda*[52]. Random forest was performed using the CRAN package *randomForest*[53], with the number of trees set to 500.

For the automatic classification of CCA sample scores, a differentiation variable was created by the difference of the mean vector of the cell subset of interest and that of all other subsets. CCA was performed using the differentiation variable, and sample scores were clustered by k-means partitioning, with *k* = 2, using the CRAN package, *cluster*[54]. A 95% confidence interval for sensitivity or accuracy was estimated as , using a jackknife estimate of the mean, *θ*[55].

## Electronic supplementary material

Additional file 1: Figure S1: Shows heatmap analysis and hierarchical clustering of the Stat3 dataset using (a) Th1, (b) Th2 (c) Th17, and (d) iTreg signatures. The cell samples that are known to differentiate into Th17 are shown by magenta. (PDF 171 KB)

Additional file 2: Figure S2: Shows the result of PCA using the Stat3 dataset. Sample relationships (sample scores) of the first 3 axes are shown. Percentage indicates that of the variance accounted for by the eigenvalue of the axis. See Colour Key for the expression values. (PDF 162 KB)

Additional file 3: Table S1: Shows CCA gene scores of the Stat3 dataset using the Th dataset for the Th differentiation programmes. (XLSX 69 KB)

## Abbreviations

CCA: Canonical Correspondence Analysis
CA: Correspondence Analysis
SVD: Singular value decomposition
PCA: Principal Component Analysis
PC: Principal Component
KNN: K-nearest neighbor
SVM: Support vector machine.
L-SVM: Linear support vector machine
NL-SVM: Non-linear support vector machine
LDA: Linear discriminant analysis
RF: Random Forest
Th: T-helper
Treg: Regulatory T cell
IL: Interleukin
IFN-γ: Interferon-gamma.
