# Supplementary figures and images for: Visualisation of the T cell differentiation programme by Canonical Correspondence Analysis of transcriptomes
Source: BMC Genomics. 2014 Nov 27;15(1):1028. doi: 10.1186/1471-2164-15-1028 (PMC4258272; doi:10.1186/1471-2164-15-1028)

Figure S1

(a) Th1 signature

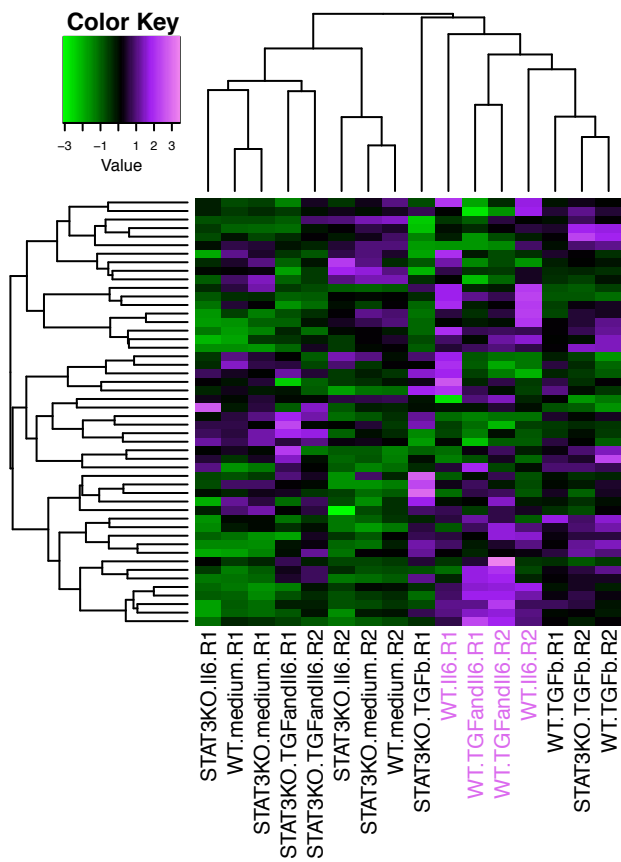

(b) Th2 signature

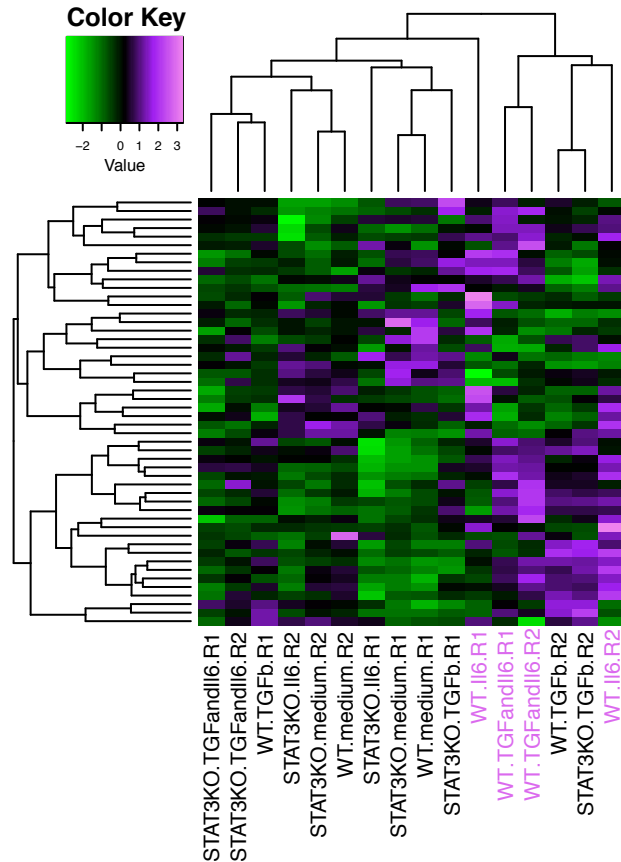

(c) Th17 signature

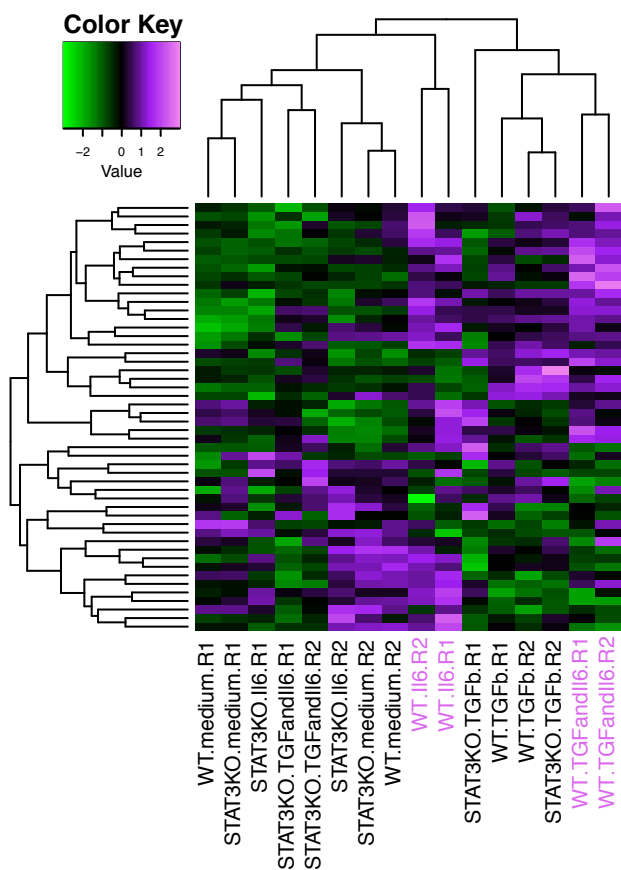

(d) iTreg signature

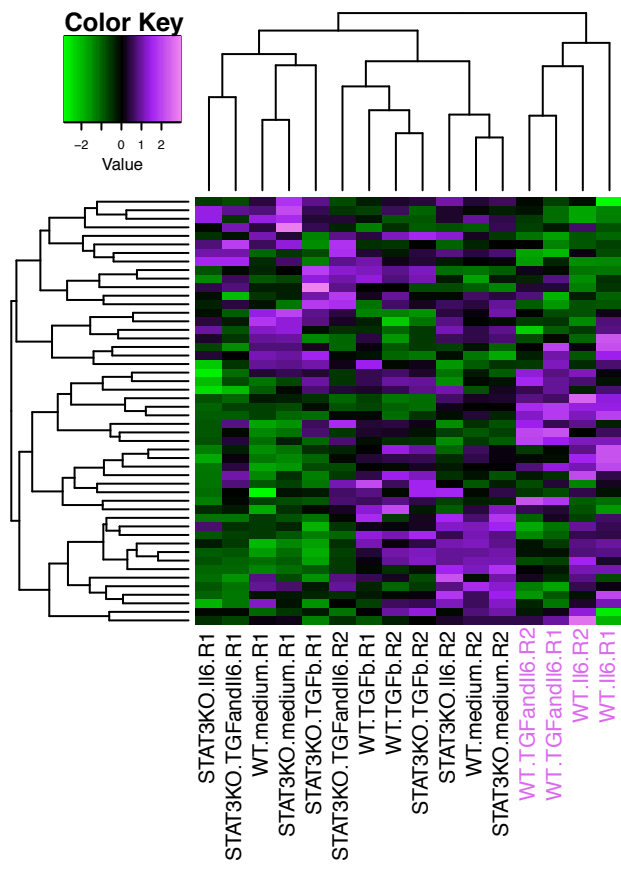

Supplement: Supplementary file 1 — Additional file 1: Figure S1: Shows heatmap analysis and hierarchical clustering of the Stat3 dataset using (a) Th1, (b) Th2 (c) Th17, and (d) iTreg signatures. The cell samples that are known to differentiate into Th17 are shown by magenta. (PDF 171 KB) [file 12864_2014_6722_MOESM1_ESM.pdf]

Figure S2

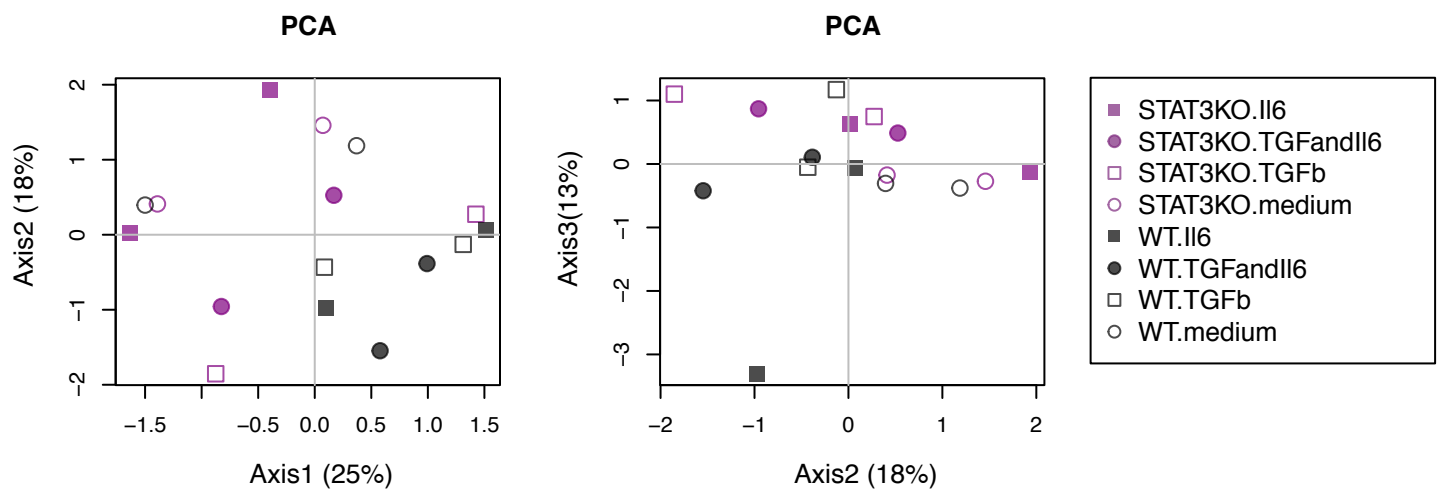

Supplement: Supplementary file 2 — Additional file 2: Figure S2: Shows the result of PCA using the Stat3 dataset. Sample relationships (sample scores) of the first 3 axes are shown. Percentage indicates that of the variance accounted for by the eigenvalue of the axis. See Colour Key for the expression values. (PDF 162 KB) [file 12864_2014_6722_MOESM2_ESM.pdf]
